# Harnessing a mesopelagic predator as a biological sampler reveals taxonomic and vertical resource partitioning among three poorly known deep-sea fishes

**DOI:** 10.1038/s41598-023-41298-9

**Published:** 2023-09-26

**Authors:** Elan J. Portner, Tor Mowatt-Larssen, Alejandro Cano-Lasso Carretero, Emily A. Contreras, Phoebe A. Woodworth-Jefcoats, Benjamin W. Frable, C. Anela Choy

**Affiliations:** 1 Scripps Institution of Oceanography, University of California San Diego, La Jolla, CA USA; 2 Virginia Institute of Marine Science, William & Mary, Gloucester Point, VA USA; 3 Cooperative Institute for Marine and Atmospheric Research, University of Hawaiʻi, Honolulu, HI USA; 4 Pacific Islands Fisheries Science Center, National Marine Fisheries Service, National Oceanic and Atmospheric Administration, Honolulu, HI USA

**Keywords:** Marine biology, Food webs, Biooceanography

## Abstract

Pelagic predators are effective biological samplers of midtrophic taxa and are especially useful in deep-sea habitats where relatively mobile taxa frequently avoid observation with conventional methods. We examined specimens sampled from the stomachs of longnose lancetfish, *Alepisaurus ferox*, to describe the diets and foraging behaviors of three common, but poorly known deep-sea fishes: the hammerjaw (*Omosudis lowii*, n = 79, 0.3–92 g), juvenile common fangtooth (*Anoplogaster cornuta*, n = 91, 0.6–22 g), and juvenile *Al. ferox* (n = 138, 0.3–744 g). Diet overlap among the three species was high, with five shared prey families accounting for 63 ± 11% of the total prey mass per species. However, distinct differences in foraging strategies and prey sizes were evident. Resource partitioning was greatest between *An. cornuta* that specialized on small (mean = 0.13 ± 0.11 g), shallow-living hyperiid amphipods and *O. lowii* that specialized on large (mean = 0.97 ± 0.45 g), deep-dwelling hatchetfishes. Juvenile *Al. ferox* foraged on a high diversity of prey from both shallow and deep habitats. We describe the foraging ecologies of three midtrophic fish competitors and demonstrate the potential for biological samplers to improve our understanding of deep-sea food webs.

## Introduction

Diets, foraging strategies, and migratory behaviors of pelagic animals vary with ontogeny and environmental conditions across trophic levels^1–3^, resulting in complex food webs. Numerous midtrophic consumers vertically migrate from daytime refuges in relatively dark mesopelagic habitats (~ 200–1000 m) to forage in epipelagic nighttime habitats (< 200 m) where primary production and total biomass are highest^4,5^. Others are non-migratory and rely on migratory prey or the passive flux of carbon from surface production to obtain food at mesopelagic depths^6,7^. However, many midtrophic fishes and squids are mobile enough to avoid sampling by nets and imaging platforms, limiting observations of their diets and foraging depths. In the deep sea, sampling avoidance has prevented robust quantification of resource partitioning among midtrophic taxa, which remains a persistent gap in our understanding of deep pelagic food webs.

Using a predator as a biological sampler of these mobile taxa can provide much needed insights into an otherwise poorly known component of deep pelagic ecosystems. Predator diet analyses, including stomach contents of fishes and squids, mammal scat, and bird boluses have been used to describe the basic biology of individual prey taxa (e.g., diet and habitat use)^8–10^. When monitored over time, predator diets can also be used to quantify how the composition and size structure of prey assemblages respond to environmental variability^11,12^. The stomachs of deep-sea fishes often function partly as a storage organ, an adaptation to food-limited habitats that preserves prey mostly undigested in stomachs and makes deep-sea fish predators exceptional candidates as biological samplers of deep-sea food webs^13,14^.

The longnose lancetfish, *Alepisaurus ferox*, occurs throughout the tropical and subtropical ocean^15,16^ and consumes a high diversity of fish, mollusk, and crustacean prey that live throughout the upper 1500 m of the water column^17–19^. *Alepisaurus ferox* exhibits ontogenetic descent from epipelagic to mesopelagic habitats between larval and adult life stages^20,21^, and increased foraging depths in individuals > 1.82 kg relative to smaller individuals^17^. In addition to feeding on poorly sampled taxa, *Al. ferox* is a useful sampler of deep-sea ecosystems because it is an abundant bycatch species on pelagic deep-set longlines^16,22^, and can be more readily collected than other deep-sea predators through partnerships with fishers and fisheries monitoring programs. Stomach content analysis of *Al. ferox* has provided several type specimens of novel species^23,24^ and has been used to study the feeding habits of mesopelagic taxa found in its stomach^25,26^.

The hammerjaw, *Omosudis lowii*, juveniles of the fangtooth, *Anoplogaster cornuta* (< 80 mm standard length, SL^27^), and juvenile *Al. ferox* (< 750 mm SL^28^) have been observed in relatively high numbers in the stomachs of large *Al. ferox* collected from the central North Pacific Ocean (CNP)^17^. All three species are midtrophic predators thought to be relatively common in pelagic ecosystems globally^13,29,30^, but are mostly able to avoid collection by trawls, with the exception of large commercial high-speed rope trawls that are infrequently used for scientific sampling in deep pelagic habitats^31^. *Anoplogaster cornuta* (max. reported size 152 mm SL^30^) exhibits diel vertical migration and occurs from ~ 135–1050 m^27^, while *O. lowii* (max. reported size 270 mm SL, this study) is largely non-migratory and found mostly at ~ 600–1000 m^21,31^. Very little is known about the feeding habits of these species in the CNP and sparse diet studies from other regions are severely limited in sample size or taxonomic resolution^26,32,33^. Although the diets of large *Al. ferox* (max. reported size 2080 mm SL^29^) are relatively well-described from several ocean basins, individuals <  ~ 400 g are also poorly studied, perhaps due to size-specific selection of hooks used on longlines^17,34^. *Omosudis lowii*, *An. cornuta* and juvenile *Al. ferox* are similarly sized, have overlapping habitats, and likely forage on a shared prey community in the CNP, but little is known about how forage resources are partitioned among them.

Using large *Al. ferox* as a biological sampler, we present a unique diet dataset to (1) describe the diets of *O. lowii*, juvenile *An. cornuta*, and juvenile *Al. ferox*, (2) quantify diet overlap with respect to taxonomic composition and size structure, and (3) evaluate how differential feeding behaviors and vertical habitat use allow for partitioning of shared resources. We also describe ontogenetic variability in the foraging depth of *Al. ferox* across body sizes spanning four orders of magnitude. This work demonstrates the importance of depth-informed diet analyses to reveal pelagic food web structure and the potential for biological samplers to expand our understanding of deep-sea ecology.

## Methods

### Specimen collection

**Figure 1:**
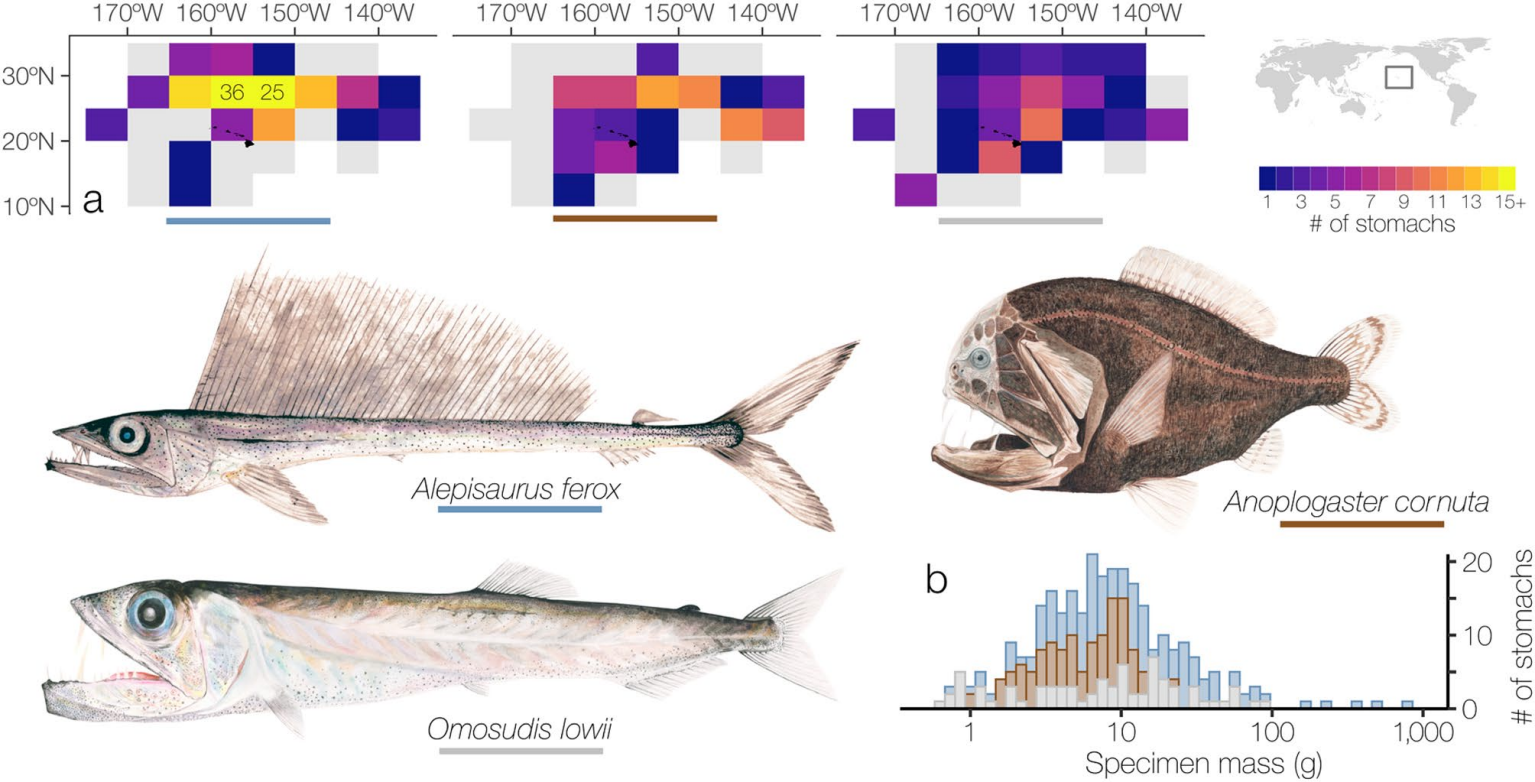
Summary of specimen size and collection location across our study area in the central North Pacific Ocean. Longnose lancetfish (*Alepisaurus ferox,* n = 138), common fangtooth (*Anoplogaster cornuta,* n = 91), and hammerjaw (*Omosudis lowii,* n = 79) were collected in the central North Pacific Ocean (**a**), mostly in waters surrounding the Hawaiian Islands. Heat maps describing the number of stomachs examined per 5° × 5° cell are overlaid on the sampling footprint for all specimens presented in this study (grey, includes primary lancetfish from Portner et al.^17^). The number of stomachs is given for cells represented by more than 14 stomachs. Specimens ranged in size from 0.32–744.90 g (**b**), but 91% of all specimens were between 1 and 100 g. Paintings by ACLC.

Lancetfish stomachs were collected by federal fisheries observers in the Hawaiʻi-based longline fishery (Hawaiʻi Longline Observer Program, https://www.fisheries.noaa.gov/inport/item/16865) from 2009 to 2020. Observers recorded fork length (FL) to the nearest centimeter as well as the date and location of capture for each specimen. Details of the collection and diet composition of longline-caught individuals, hereafter referred to as “primary lancetfish”, are described in Choy et al.^35^ and Portner et al.^17^. *Alepisaurus ferox* (n = 138), *An. cornuta* (n = 91), and *O. lowii* (n = 78) were opportunistically sampled from the stomachs of primary lancetfish for further diet study and are presented here for the first time (Fig. 1, Fig. S1). An additional *O. lowii* was directly sampled by longline observers and included in the analyses (Table 1). In most cases, specimens were not processed immediately and were re-frozen at − 20 °C. Thawed specimens were weighed (nearest 0.01 g) and measured (SL to the nearest 1 mm) before their stomachs were dissected. Prey were identified to the finest possible taxonomic resolution and assigned a digestion state (following Choy et al.^35^, 1 = completely intact, 2 = minimally digested, 3 = partially digested, and 4 = heavily digested). Groups of prey with the same taxonomic ID and digestion state in a single stomach (“prey group”) were enumerated and weighed. Stomach content mass was subtracted from whole specimen mass to obtain predator masses used in the analyses. Although we present some of the largest known diet data sets for these deep-sea fishes, sample sizes were not large enough to explore spatial or temporal variability in diets across our study area. The spatial and temporal coverage of sampling was similar among species (Fig. 1, Fig. S1) and stomachs of each predator were pooled across sampling years and locations for all analyses. Unless otherwise specified, analyses were performed with packages in R Statistical Software (version 3.6.3^36^).

**Table 1:** Summary of specimen sizes and sample numbers per species.

	n	SL range (mm) Mean (± sd)	Mass range (g) Mean (± sd)	*n*_*wp*_ (%n)	*n*_*sa*_ (%*n*_*wp*_)	*n*_*fd*_ (%*n*_*wp*_)
*Alepisaurus ferox*	138	36–700 196.02 (110.01)	0.33–744.90 28.24 (82.57)	116 (84.06)	102 (87.93)	106 (87.07)
*Anoplogaster cornuta*	91	25–78 53.33 (13.92)	0.56–22.08 6.31 (3.96)	81 (89.01)	73 (90.12)	74 (91.36)
*Omosudis lowii*	79	43–270 123.76 (55.68)	0.32–92.00 13.27 (17.64)	40 (50.63)	30 (75.00)	36 (87.50)

Minima, maxima, means and standard deviations of the specimen lengths (standard length, SL mm) and masses (g) are given for each species. The total number of stomachs examined (n), as well as the number of stomachs with prey (*n*_*wp*_) included in diet similarity (*n*_*sa*_) and foraging depth analyses (*n*_*fd*_) are also given.

### Diet composition analyses

Diet composition was quantified as the total abundance, mass, and frequency of occurrence for each prey group. The mean proportional abundance ($\overline{N}$) and mass ($\overline{M}$) per stomach (prey group total/stomach total), as well as the percent frequency of occurrence per predator (%*FO*) were also quantified. Prior to analyses, $\overline{M}$ was recalculated at the family level to ensure all prey group identifications were mutually exclusive and reduce the number of zeros in the diet matrix^37^. Four prey groups considered mutually exclusive from all family-level groups were included at a coarser taxonomic resolution in the family-level analyses. Hyperiid amphipods in the families Brachyscelidae and Lycaeidae were conservatively lumped together into the superfamily Platysceloidea. Crustacean megalopa, fish leptocephali, and deep-sea anglerfishes (Ceratioidei) were not identified further but were each exclusively found in the stomachs of a single predator species and could thus reliably describe diet variability among species. To reduce potential influence of variable predator size distributions on diet comparisons, diet overlap analyses were limited to specimens between 1 and 100 g (91% of specimens). No size restrictions were applied to analyses that explicitly account for predator mass.

Diet overlap among species was quantified using analysis of similarities (ANOSIM) in Primer v.7^38^. Pairwise tests for homogeneity of multivariate dispersion (PERMDISP) and permutational multivariate analysis of variance (PERMANOVA) among species were also performed in Primer v.7. To assess contributions of centroid location and dispersion on observed differences among species, diet overlap was visualized using NMDS in the *vegan* package (version 2.5-7^39^). In all cases, analyses were performed on a matrix of Morisita-Horn similarities (*C*_*mh*_^40^). To reduce the influence of rare taxa on distance metrics (e.g., Legendre & Gallagher^41^), only prey families contributing more than 1%$\overline{M}$ for at least one predator species and found in more than one stomach were included in similarity analyses. Unidentified prey groups were also excluded. Family-level sample coverage and diet diversity (Shannon diversity (^1^*D*), Hill number of order q = 1^42^) were quantified using the *iNEXT* package (version 2.0.20^43^). True diversity was estimated as the asymptotic Shannon diversity (^1^*D*_*ex*_) and the number of samples required to reach 95% sample coverage are reported to describe the rate of diversity accumulation.

To examine differences in feeding strategies among predators, %*FO* and prey-specific proportional mass (%$\overline{M}$_*ps*_*)* were quantified for the four most important prey groups for each predator. Prey importance was quantified as the index of relative importance ((%$\overline{N}$ + %$\overline{M}$)* %*FO*^44^)*. *%$\overline{M}$_*ps*_ is a modified proportional mass metric calculated using only stomachs that contained the prey of interest, an analog of the abundance-based metric described in Amundsen et al.^45^. A 50%$\overline{M}$_*ps*_ threshold can be used to distinguish specialist (> 50) and generalist (< 50) feeding, and when combined with a 50%*FO* threshold can help describe how prevalent the feeding strategy is among individuals^45^.

To facilitate comparisons with previous studies, we recalculated $\overline{M}$ for broad prey types and examined ontogenetic changes in diet contributions. Variability in the $\overline{M}$ contributions of fish, mollusk, and crustacean prey with predator size were assessed separately for each predator species by fitting generalized additive models (GAMs) specified with beta error distributions and “logit” link functions. All GAMs presented in this study were fit using restricted maximum likelihood estimation in the *mgcv* package (version 1.8-38^46^). A single model specified with a beta error distribution describing $\overline{M}$ of prey in *O. lowii* was dominated by zeros and ones and would not converge. To improve model performance, we excluded stomachs containing more than one prey type (n = 2, retaining 95% of *O. lowii* specimens with prey) and fit a GAM specified with a binomial error distribution and “logit” link function.

### Prey mass, length, and abundance

For prey groups with digestion states 1–3, individuals were weighed (nearest 0.01 g) and measured (nearest mm; SL for fishes, mantle lengths (ML) for cephalopods, and total lengths for crustaceans). If a prey group contained more than three individuals, a subset representing the minimum, median, and maximum sizes were qualitatively selected and measured. For unmeasured prey items in each stomach, individual mass and length were estimated as the median mass and length of measured individuals from the same prey group. If no individuals of a given prey group were measured, individual mass was estimated by dividing total prey group mass by the number of individuals. GAMs describing family-level length-to-mass relationships for measured individuals were specified with gaussian error distributions and “identity” link functions. Estimated prey lengths for unmeasured individuals were predicted based on estimated prey masses using the family-level GAMs.

The effects of predator species and mass on individual prey mass, total prey mass per stomach, and total number of prey per stomach were examined using multilinear models in R. To meet model assumptions, prey mass, total prey mass, and predator mass were log_10_-transformed and prey count was log_2_-transformed prior to model fitting (estimated using ordinary least squares regression). Ninety-five percent confidence intervals and *p *values were computed using a Wald *t*-distribution approximation. Model assumptions were checked using standard diagnostic plots in R. The relative explanatory power of predator species and mass in each model was quantified using analysis of covariance (ANCOVA, type III sum of squares) on model outputs in the *car* package (version 3.0-13^47^). Statistics describing individual prey mass only include prey in digestive states 1–3. Total prey masses and counts reflect all prey items in a single stomach, regardless of digestion state or level of taxonomic identification.

### Foraging depths

To examine variability in foraging depths among predator species and across sizes, the foraging depth of each predator was estimated as the weighted median depth of occurrence of prey in its stomach (Eq. 1). Only prey taxa in families contributing at least 1%$\overline{M}$ to the overall diet composition for any predator species were included in foraging depth estimations. Median depth of occurrence for each prey taxon was assigned based on reported depths from the literature (Table S1). For taxa that exhibit diel vertical migration, median depths were assigned as the mean of reported daytime and nighttime median depths. For taxa known to exhibit variable habitat depths across ontogeny, length-specific median habitat depths were assigned. For genus- and family-level identifications, median depths of occurrence were averaged from multiple congeners and representative confamilials. The proportional mass of each prey taxa was recalculated using an adjusted total mass per stomach that only included prey for which depth data were available (_adj_$\overline{M}$). Predator foraging depth (*Z*_*f*_) was estimated based on all prey taxa in a single stomach (*n*) as a function of the median depth of occurrence (*z*) and adjusted proportional mass (_adj_$\overline{M}$) of each prey taxa (*i*):

1 $$Z_{f} = \sum\limits_{i}^{n} ({{\text{z}}_{{{\text{i}}}}} \times {_{{\text{adj}}}} \overline{M}_{i})$$

Differences in estimated foraging depths among predator species and across sizes were assessed by fitting a GAM specified with a gaussian error distribution and identity link function.

### Ontogeny of predator consumption by lancetfish

To more directly link ontogenetic variability in lancetfish foraging depth to the consumption of *Al. ferox*, *An. cornuta*, and *O. lowii*, diet data from *n* = 1066 primary lancetfish presented in Portner et al.^17^ were reanalyzed as described above with some notable differences. Primary lancetfish specimen mass was estimated from FL using a published regression^48^. Polychaetes in the tribe Alciopini and heteropods in the family Carinariidae are relatively fragile taxa that were common in primary lancetfish stomachs but rarely intact, regardless of digestion state. These prey groups were never individually measured but are best represented by epipelagic taxa and were assigned to a single depth habitat regardless of estimated size (Table S1). For cephalopods in families contributing at least 1%$\overline{M}$, masses and MLs of individuals identified from beaks were estimated using published regressions following Chen et al.^49^ and included in _adj_$\overline{M}$ calculations.

Differences in foraging depths across predator species and sizes were re-assessed after including primary lancetfish data by fitting a GAM specified with a gaussian error distribution and “identity” link function. To determine how well foraging depths estimated from stomach contents reflect known habitat usage, estimates were compared to reported median depths of occurrence for each predator (Table S1). The estimated foraging depths of all three species, including primary lancetfish, were qualitatively compared to changes in the %*FO* of all three species in the stomachs of *Al. ferox* with increasing size.

## Results

### Diet description and overlap among predators

**Figure 2:**
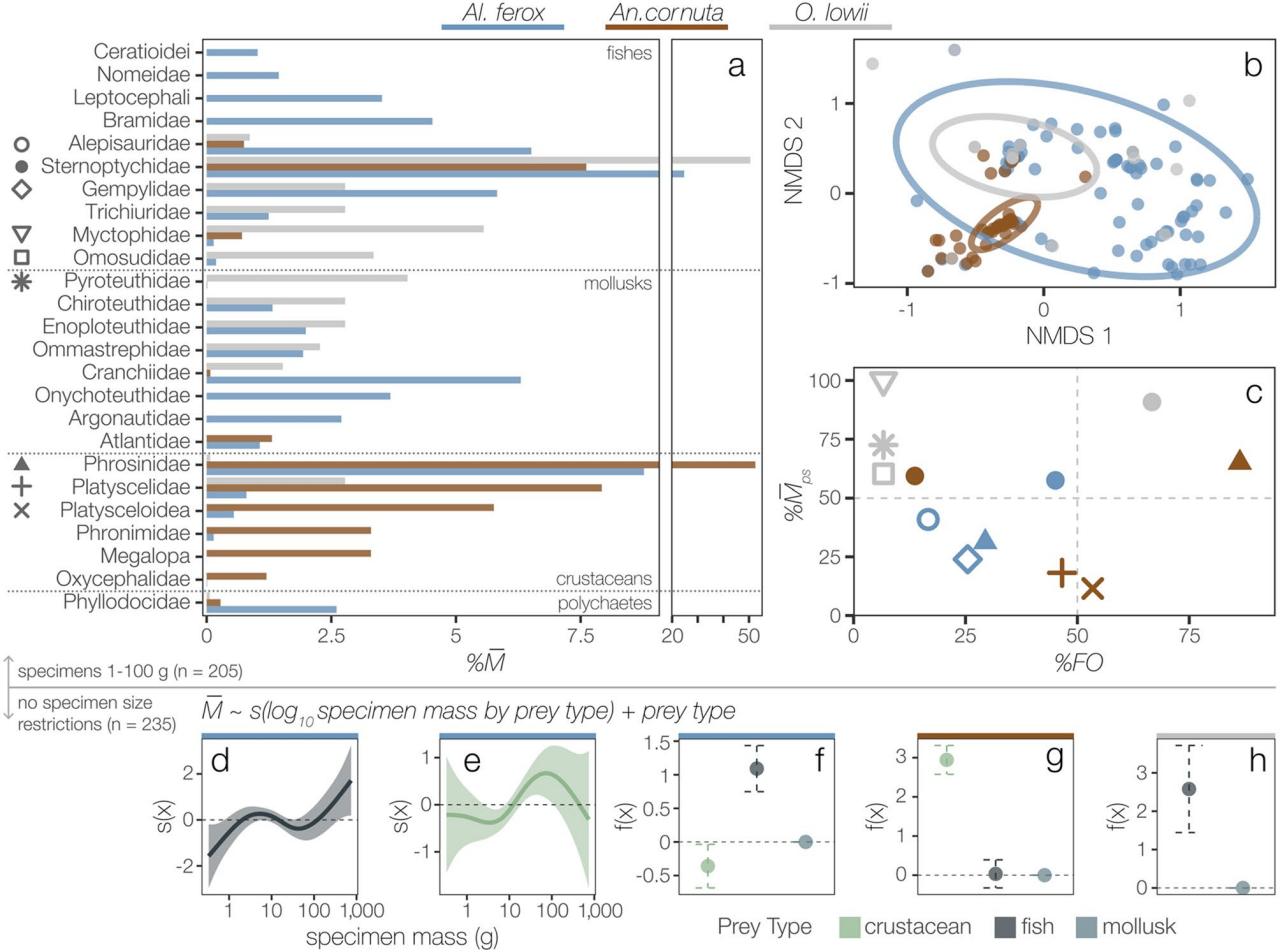
Diet overlap is high among predator species, but there is consistent taxonomic resource partitioning. (**a**–**c**) Family-level diet composition and overlap among predators 1–100 g for prey families contributing > 1% mean proportional mass (%$\overline{M}$). (**a**) *Alepisaurus ferox* (n = 102) consumed a high diversity of fish, crustacean, and mollusk families, while *Anoplogaster cornuta* (n = 73) and *Omosudis lowii* (n = 30) diets were dominated by crustacean and fish families, respectively. The x-axis is broken into two scales to improve visualization. (**b**) The first two NMDS axes (stress = 0.096, RMSE = 0.001) and 95% confidence interval ellipses depict relatively high diet overlap between *Al. ferox* and the other two species, and low overlap between *An. cornuta* and *O. lowii*. (**c**) Percent frequency of occurrence (%*FO*) and %$\overline{M}$ recalculated just for stomachs containing the prey family (prey-specific mean proportional mass, %$\overline{M}$_*ps*_) are given for the four most important prey families for each predator. Dashed lines distinguish specialist (> 50%$\overline{M}$_*ps*_) from generalist (< 50%$\overline{M}$_*ps*_) feeding strategies at individual (< 50%*FO*) and population levels (> 50%*FO*). Shapes represent the corresponding families in panel (**a**). (**d**–**h**) Partial effects plots from generalized additive models describe changes in the $\overline{M}$ of prey types with predator size for each species, where axes describe the relationship between a covariate and its parametric contribution (“f(x)”) or the contribution of its smoother (“s(x)”) to the model’s fitted values. *Alepisaurus ferox* is increasingly piscivorous with size (n = 116) (**d**–**f**), while the prey type preferences of *An. cornuta* (n = 81) (**g**) and *O. lowii* (n = 38) (**h**) did not vary across the sizes examined. Model summaries and partial effects plots for all covariates are given in Table 2 and Fig. S3, respectively.

Of the 308 predator stomachs examined, 116 *Al. ferox* (84%), 81 *An. cornuta* (89%), and 40 *O. lowii* (51%) contained prey. A total of 2035 prey individuals representing 113 unique taxa were identified from 58 families (32 fish, 16 mollusk, 8 crustacean, and 2 other invertebrates). Counts, masses, frequency of occurrence, and proportional metrics (%$\overline{N}$, %$\overline{M}$, %*FO*) of each prey type are given for each predator in Table S2. After removing predators < 1 or > 100 g, unidentified prey groups, and families contributing < 1%$\overline{M}$, 102 *Al. ferox*, 73 *An. cornuta*, and 30 *O. lowii* were included in diet similarity analyses. Twenty-five prey families contributed at least 1%$\overline{M}$ (Fig. 2a) and accounted for 88 ± 19% of the total prey mass in each stomach. The most important prey across all predators were hatchetfishes (Sternoptychidae) in the genus *Sternoptyx* and the hyperiid amphipod, *Phrosina semilunata* (Phrosinidae). Only five prey taxa were shared among all three predators, but accounted for 50%, 71%, and 67% of the total prey mass in *Al. ferox*, *An. cornuta*, and *O. lowii* stomachs, respectively (Table S2).

*Alepisaurus ferox* diets were the most diverse (^1^*D*_*ex*_ = 17.57 ± 0.90, Table S3, Fig. 2b, Fig. S2) and contained all unique prey types (Table S2). The four most important prey groups (amphipods in the family Phrosinidae and fishes in the families Sternoptychidae, Alepisauridae, and Gempylidae) were consumed with moderate frequency and mostly with low %$\overline{M}$_*ps*_, reflecting a more generalist feeding strategy (Fig. 2c). *Anoplogaster cornuta* diets were dominated by crustaceans and exhibited a high degree of specialization on Phrosinidae (65%$\overline{M}$_*ps*_, 86%*FO*). Platyscelid and platysceloid hyperiid amphipods were also observed at relatively high frequencies and each stomach contained a high proportion of the total observed diversity (*t*_95%_ = 12, Table S3). When present, Sternoptychidae comprised most of the stomach content mass for all predators (> 50%$\overline{M}$_*ps*_) but dominated the diets of *O. lowii* (91%$\overline{M}$_*ps*_, 67%*FO,* Fig. 2c). *Omosudis lowii* had similar diet diversity to *An. cornuta* (^1^*D*_*ex*_ = 9.33 ± 3.08 and 7.88 ± 0.37, respectively), but exhibited the lowest rate of family-level prey diversity accumulation (*t*_95%_ = 113). Diets were significantly different among predators (ANOSIM, *Global R* = 0.27, *p* < 0.001). Pairwise differences among predators could be explained by differences in both mean diet composition (PERMANOVA, Table S4) and variance (Fig. 2b, PERMDISP, *F* = 44.36, *p* < 0.001, Table S4). Diets of *O. lowii* were most similar to *Al. ferox* (*C*_*mh*_ = 22.44) and least similar to *An. cornuta* (*C*_*mh*_ = 6.71, Fig. 2b).

There was limited variability in the relative mass contributions of broad prey types (mollusk, fish, and crustacean) to diet composition with increasing size for all predators (Table 2). *Alepisaurus ferox* became increasingly piscivorous with size and exhibited an increase in the $\overline{M}$ of crustacean prey across intermediate sizes (~ 10–100 g, Fig. 2d–f). *Anoplogaster cornuta* diet was dominated by crustaceans regardless of size and there was no clear variability in the relative contributions of prey types across the sizes examined (Fig. 2g). *Omosudis lowii* was strongly piscivorous across all sizes examined. The $\overline{M}$ of fish prey was two- to three-times larger than the $\overline{M}$ of mollusk prey on average (Fig. 2h), and there were no changes in the relative contributions of fish and mollusk prey with *O. lowii* size (Fig. S3).

**Table 2:** Covariate contributions to generalized additive models describing changes in the proportional mass ($\overline{M}$) of broad prey types with predator mass (*a, b, c*) and the effects of predator species and mass on estimated foraging depth (*d, e*).

**(a)** ***Alepisaurus*** $\overline{\varvec{M}}$ **~ s(log**_**10**_**(predator mass), by prey type) + prey type, n = 116, *Adj. R***^***2***^** = 0.30**				
Parametric coefficients	est	se	*z*	*p*
(Intercept)	− 0.90	0.12	− 7.54	4.87 E^−14^
*Fish* vs. *Mollusk*	1.09	0.17	6.24	4.31 E^−10^
*Crustacea* vs *Mollusk*	− 0.36	0.17	− 2.17	0.03
*Crustacea* vs. *Fish*	− 1.45	0.17	− 8.48	< 2.00 E^−16^

Smooth terms	edf		*Χ*^2^	*p*
*s(predator mass): Mollusk*	1.00		0.70	0.40
*s(predator mass): Fish*	3.74		13.72	0.01
*s(predator mass): Crustacea*	3.33		10.94	0.03

**(b)** ***Anoplogaster,*** $\overline{\varvec{M}}$ **~ s(log**_**10**_**(predator mass), by prey type) + prey type, n = 81, *Adj. R***^***2***^** = 0.67**				
Parametric coefficients	est	se	*z*	*p*
(Intercept)	− 1.68	0.13	− 12.99	< 2.00 E^−16^
*Fish* vs. *Mollusk*	0.03	0.18	0.18	0.86
*Crustacea* vs *Mollusk*	2.94	0.19	15.71	< 2.00 E^−16^
*Crustacea* vs. *Fish*	2.91	0.19	15.50	< 2.00 E^−16^

Smooth terms	edf		*Χ*^2^	*p*
*s(predator mass): Mollusk*	1.00		0.29	0.59
*s(predator mass): Fish*	1.01		0.52	0.47
*s(predator mass): Crustacea*	1.00		0.00	0.97

**(c)** ***Omosudis,*** $\overline{\textbf{\textit{M}}}$ **~ s(log**_**10**_**(predator mass), by prey type) + prey type, n = 38, *Adj. R***^***2***^** = 0.34**				
Parametric coefficients	est	se	*z*	*p*
(Intercept)	− 1.35	0.41	− 3.32	8.98 E^−04^
*Fish* vs. *Mollusk*	2.58	0.58	4.46	8.34 E^−06^

Smooth terms	edf		*Χ*^2^	*p*
*s(predator mass): Mollusk*	1.66		1.35	0.49
*s(predator mass): Fish*	2.96		4.47	0.39

**(d) Foraging depth ~ s(log**_**10**_**(predator mass), by prey type) + prey type, n = 216, *Adj. R***^***2***^** = 0.22**				
Parametric coefficients	est	se	*t*	*p*
(Intercept)	346.12	19.21	18.01	< 2.00 E^−16^
*Anoplogaster* vs. *Alepisaurus*	− 143.74	34.19	− 4.20	3.89 E^−05^
*Omosudis* vs *Alepisaurus*	148.65	37.55	3.96	1.03 E^−04^
*Omosudis* vs. *Anoplogaster*	292.39	42.90	6.82	9.96 E^−11^

Smooth terms	edf		*F*	*p*
*s(predator mass):Alepisaurus*	3.08		1.33	0.35
*s(predator mass):Anoplogaster*	1.00		0.84	0.36
*s(predator mass):Omosudis*	1.00		5.51	0.02

**(e) Foraging depth ~ s(log**_**10**_**(predator mass), by species) + species, n = 1220, *Adj. R***^***2***^** = 0.12**				
Parametric coefficients	est	se	*t*	*p*
(Intercept)	464.52	8.80	52.79	< 2.00 E^−16^
*Anoplogaster* vs. *Alepisaurus*	− 405.458	257.04	− 1.58	0.11
*Omosudis* vs *Alepisaurus*	309.30	174.86	1.77	0.08
*Omosudis* vs. *Anoplogaster*	714.77	310.62	2.30	0.02

Smooth terms	edf		*F*	*p*
*s(predator mass):Alepisaurus*	3.21		28.73	< 2.00 E^−16^
*s(predator mass):Anoplogaster*	1.00		0.39	0.53
*s(predator mass):Omosudis*	1.00		2.57	0.11

Coefficient estimates (“est.”), standard error (se), *z*-values, and *p*-values are given for each parametric term. Estimated degrees of freedom (edf), Chi-squared (*Χ*^2^), and *p*-values are given for each smooth term. Partial effects plots for covariates with *p*-values < 0.05 from *a–c* are given in Fig. 2. Partial effects plots for all covariates from each model are given in Fig. S3.

### Size-based diet partitioning and total prey consumption

**Figure 3:**
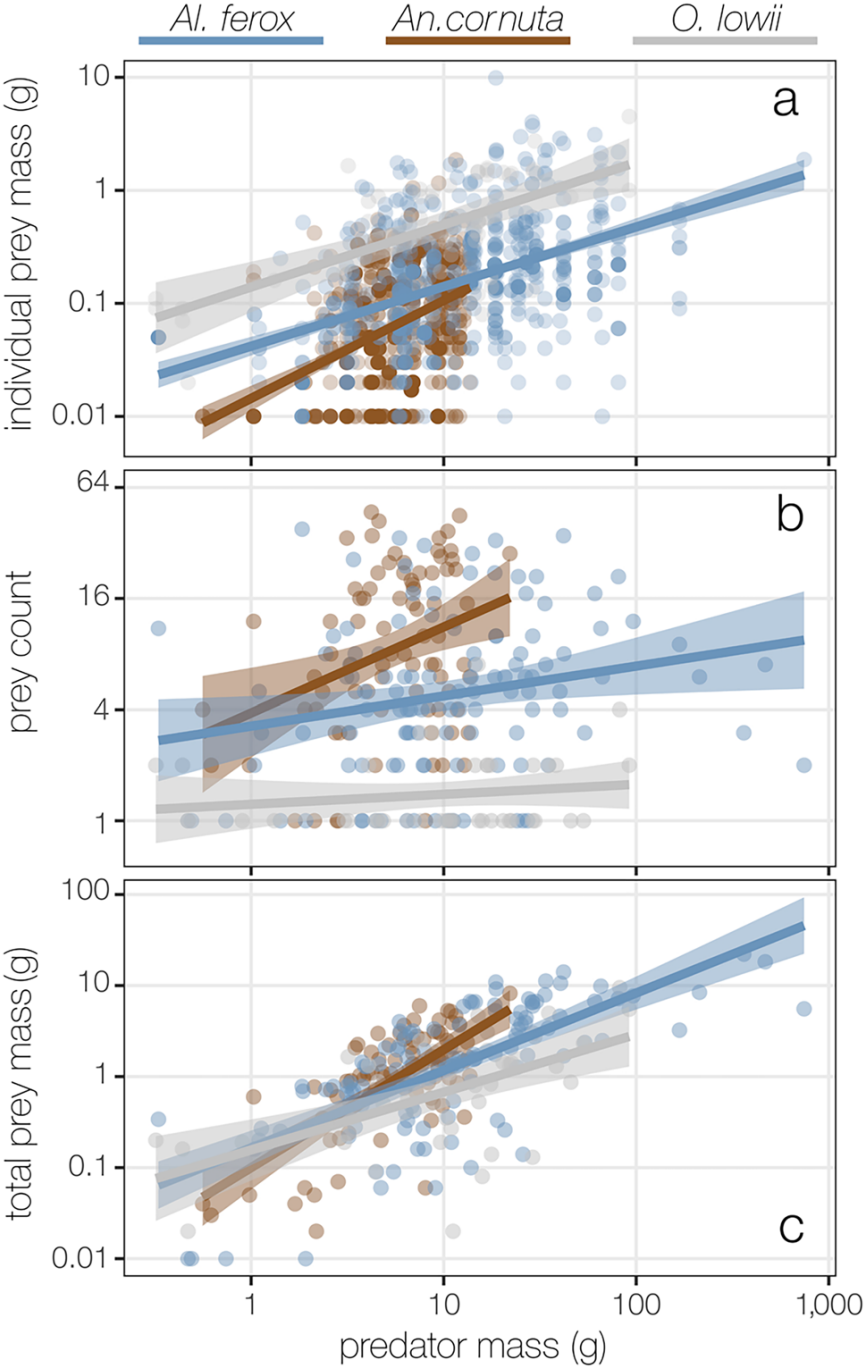
Differences in the individual size and number of prey per stomach result in similar total prey mass among predators. Multi-linear regressions describing changes in individual prey size (n = 1608) and the total amount of prey per stomach (n = 237) with increasing predator mass. All metrics increased with predator mass, but although there were differences in the mass of individual prey (**a**) and total prey count per stomach (**b**) among predator species, there was no difference in the total prey mass per stomach among species (**c**) when acounting for predator mass. Y-axes are on the log_10_-scale in panels (**a**) and (**c**) and on a log_2_-scale in panel (**b**). Linear models and ANCOVA results for each model are given in Table 3 and model summaries are given in Table S6.

Individual prey mass varied among predators and increased with predator mass (Fig. 3a; *adj. multiple R*^2^ = 0.30, *F*(5, 1602) = 137.70, *p* < 2.20 E^−16^; Table 3, Table S5). *Omosudis lowii* consumed larger prey than either *Al. ferox* or *An. cornuta* across all predator sizes. Trends in prey size differences among predator species were largely consistent across prey types (Fig. S4a–c; Table S6). However, differences in the sizes of the dominant shared prey group, Sternoptychidae, were not statistically clear among predators or across predator sizes for *An. cornuta* or *O. lowii* (Fig. S4e, Table S6). Although *Al. ferox* also consumed larger prey than *An. cornuta*, the difference in average prey size decreased with increasing predator mass (Fig. 3a), driven by increased crustacean prey size in *An. cornuta* (Fig. S4a,d).

**Table 3:** Model results and ANCOVA summaries for all multilinear models describing the effects of predator species and mass on stomach contents and estimated foraging depth.

	*SS*	*df*	*F*	*p*
**(a) log**_**10**_**(individual prey mass) ~ log**_**10**_(predator mass)*species, *F*(5, 1602) = 137.86, *p* < 2.2 E^**−16**^**, *adj.R***^***2***^** = 0.30**				
Intercept	234.53	1	1084.56	< 2.20 E^−16^
*log*_10_*(predator mass)*	34.80	1	160.95	< 2.20 E^−16^
*Species*	15.13	2	34.99	1.33 E^−15^
*log*_10_*(predator mass):species*	4.15	2	9.59	7.21 E^−05^
Residuals	346.42	1602		
**(b) log**_**2**_** (pre﻿y count) ~ log**_**10**_(predator mass)*species, *F*(5, 231) = 23.57, *p* < 2.2 E^**−16**^**, *adj. R***^***2***^** = 0.32**				
Intercept	46.70	1	26.11	6.77 E^−07^
*log*_10_*(predator mass)*	18.65	1	10.43	1.42 E^−03^
*Species*	18.39	2	5.14	6.55 E^−03^
*log*_10_*(predator mass):species*	9.50	2	2.65	0.07
Residuals	413.23	231		
**(c) log**_**10**_**(total prey mass) ~ log**_**10**_(predator mass)*species, *F*(5, 231) = 41.78, *p* < 2.2 E^**−16**^**, *adj. R***^***2***^*** = 0.46***				
Intercept	12.38	1	52.67	5.96 E^−12^
*log*_10_*(predator n mass)*	13.13	1	55.83	1.62 E^−12^
*Species*	0.42	2	0.89	0.41
*log*_10_*(predator mass):species*	2.17	2	4.61	0.01
Residuals	54.31	231		

The sum of squares (*SS*), degrees of freedom (*df*), *F*-statistics (*F*) and *p*-values (*p*) are given for terms and their interactions (*:*) for each model.

Predator species and mass were both significant predictors of prey counts per stomach (Fig. 3b; *adj. multiple R*^2^ = 0.32, *F*(5, 231) = 23.57, *p* < 2.20 E^−16^; Table 3). There was no difference in the number of prey per stomach between *Al. ferox* and *An. cornuta,* but both consumed more prey individuals on average than *O. lowii* (Fig. 3b, Table S6). All predators consumed more prey with increasing size, but the difference in prey counts per stomach between *An. cornuta* and *O. lowii* increased with predator size.

The total mass of prey per stomach increased with predator mass (Fig. 3c; *adj. multiple R*^2^ = 0.46, *F*(5, 231) = 41.78, *p* < 2.20 E^−16^), but was not clearly different among predator species (Table 3). The interaction effect of predator species and mass on total prey mass is statistically significant but weak (ANCOVA, *F* = 4.61, *p* = 0.01), driven by differences in the interaction term for *An. cornuta* compared to both *Al. ferox* an *O. lowii* (lm, *t* = (− 2.34, − 3.03), *p* = (0.02, 0.003), respectively, Table S5).

### Foraging depths and changes in overlap with predator size

**Figure 4:**
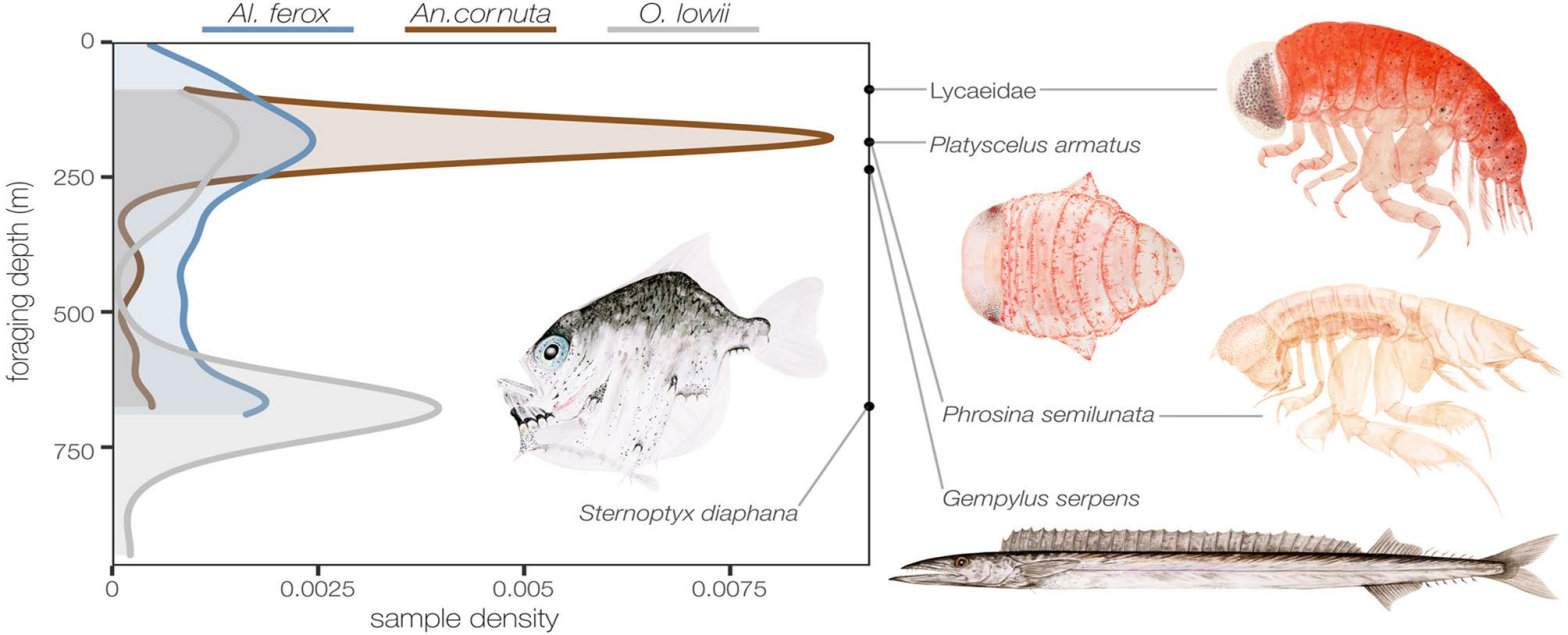
Foraging depths reflect differential vertical resource use among predators. Foraging depths were estimated for each predator (n = 216) as the weighted median depth of occurrence of all prey in a single stomach that had been identified at least to family and contributed more than 1% mean proportional abundance. *Anoplogaster cornuta* fed mostly on hyperiid amphipods with relatively shallow median depths of occurence (e.g., Lycaeidae 87.5 m, *Platyscelus armatus* 185 m, *Phrosina semilunata* 185.38 m; Table S1), while *Omosudis lowii* fed mostly on deep-dwelling hatchetfishes (e.g., *Sternoptyx diaphana* 675 m). *Alepisaurus ferox* foraged more evenly across the upper 700 m of the water column on shared prey, but also incorporated a higher diversity of prey in their shared habitats (e.g., *Gempylus serpens* 237 m). Paintings by ACLC.

Estimated foraging depths were clearly different among predator species (*n* = 106 *Al. ferox*, *n* = 74 *An. cornuta*, and *n* = 36 *O. lowii*, Table 2) and were quantified using an average 89 ± 8% of the total prey mass per stomach across species. Based on weighted median depths of prey occurrence, the predators foraged in very different depth habitats, mostly either ~ 180 or ~ 675 m (Fig. 4). These depths correlate with the median depths of the two most important prey taxa (*Phrosina semilunata* and *Sternoptyx* spp., respectively, Table S1), but also align with the maxima of the bimodal distribution of median depths for all reported prey taxa (Fig. S5).

*Omosudis lowii* foraged deepest (GAM, intercept = 494.77 m, 95%* CI  *[421.17, 568.37], *p* < 0.001, Table 2), driven by its consumption of hatchetfishes in the genus *Sternoptyx* spp., a non-migratory group with a median depth of occurrence of 675 m (Fig. 4). *Anoplogaster cornuta* had the shallowest mean estimated foraging depth (GAM, intercept = 202.38 m, 95%* CI* [135.37, 269.39], *p* < 0.001), consuming large quantities of hyperiid amphipods in six families with median depths of occurrence in the upper 250 m. *Alepisaurus ferox* fed in both shallow and deep habitats and more broadly throughout the intervening water column (GAM, intercept = 346.12 m, 95%* CI* [308.47, 383.77], *p* < 0.001; Fig. 4). Predator size was not a useful predictor of foraging depth for *Al. ferox* or *An. cornuta,* but the foraging depth of *O. lowii* increased with size (GAM, *F* = 5.51,* p* = 0.02).

**Figure 5:**
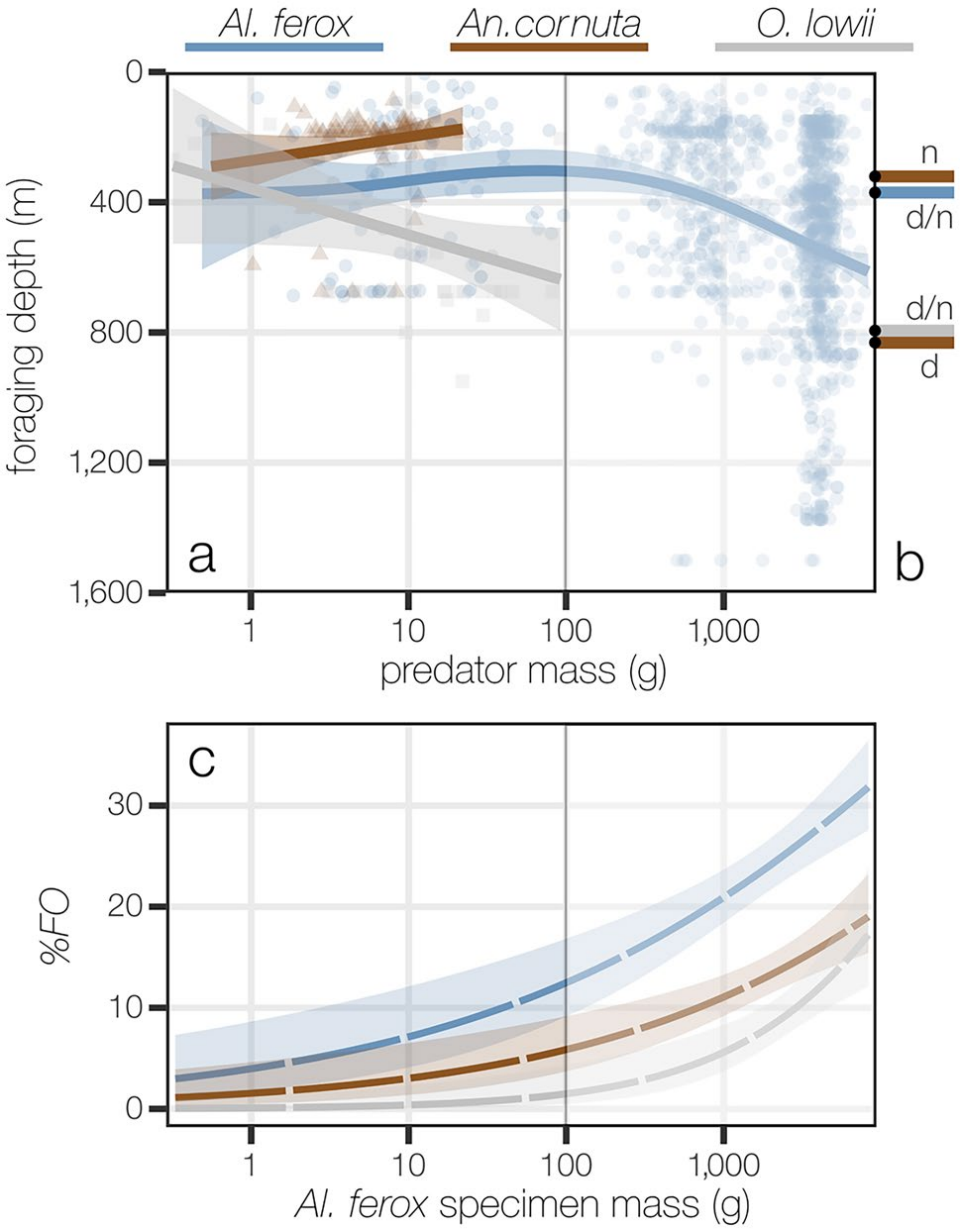
Vertical habitat overlap among predator species varies with size, and predator consumption by *Alepisaurus ferox* increases with foraging depth. The foraging depths of *Anoplogaster cornuta* and *Al. ferox* did not vary with mass, while the foraging depth of *Omosudis lowii* increased with mass across the size of specimens examined in this study (**a**). When primary lancetfish from Portner et al.^17^ were included [>99% of specimens larger than 100 g, indicated by vertical line and shading in panels (**a**) and (**c**)], overlap in foraging depths between *Al. ferox* and *O. lowii* increased with *Al. ferox* mass. Regression lines in panel (**a**) were fit with generalized additive models (GAM) for each predator species. Partial effects plots for the full GAMs with and without primary lancetfish as described in Table 2 are given in Fig. S3. The median reported habitat depths of each predator (**b**) during day (“d”) and night (“n”), represented as bars extending from the right-hand y axis of panel (**a**), were very similar to the foraging depths estimated in this study. Only *An.* *cornuta* is known to undergo diel vertical migration. Regressions fit with generalized linear models using a binomial error distribution describe increased frequency of occurrence of all three predator species in the stomach of *Al. ferox* with specimen mass (**c**).

Of the 1066 primary lancetfish containing prey, foraging depths could be estimated for 1004 specimens using an average of 89 ± 21% of the total prey mass per stomach. When these specimens are considered, the average foraging depth of *Al. ferox* increases with size (Fig. 5a, GAM, *F* = 28.73, *p* < 2.2E^−16^, Table 2). Overlap in foraging depths between *Al. ferox* and *O. lowii* also increased with size and there was no discernable difference in foraging depths between them once large *Al. ferox* (> ~ 4 kg) were included. The average foraging depths estimated from stomach contents are very similar to the median depths of occurrence reported for all three predators (Fig. 5b, Table S1). Estimated foraging depths for most *An. cornuta* match reported nighttime median depths of occurrence (160–375 m)^27^. Eleven percent of *An. cornuta* stomachs contained *Sternoptyx* spp., which were likely consumed closer to the predator’s reported daytime median depths of occurrence (650–950 m).

The %*FO* of *Al. ferox*, *An. cornuta*, and *O. lowii* prey were also quantified for the 1004 primary lancetfish with estimated foraging depths. As prey, the frequency of occurrence of all three species increased with *Al. ferox* size. The incidence of cannibalism increased from 5%*FO* in the smallest individuals to 35%*FO* in the largest, and cannibalism was more frequent than the consumption of *An. cornuta* or *O. lowii* across all sizes of *Al. ferox* (Fig. 5c). However, the %*FO* of *O. lowii* in the stomachs of *Al. ferox* was near zero for specimens < 100 g and increased rapidly in specimens > 1 kg as overlap in foraging depths between the two species increased.

## Discussion

Stomach content analysis of *Al. ferox* is well-suited to address a practical gap in our sampling of mobile midtrophic taxa in pelagic ecosystems, providing specimens of poorly sampled fauna from shallow and deep-sea habitats in suitable condition for diet analyses. Additionally, there is a rich literature describing the depth habitats of small organisms that comprise the forage base for larger midtrophic taxa found in the stomachs of *Al. ferox*. By using observations of prey vertical habitats to infer predator foraging depths, we describe how variability in foraging ecology and resource overlap among three mesopelagic fishes is mediated by ontogenetic variability in habitat use. Although less specific than observations made with depth-discrete trawls for describing vertical habitats, this work demonstrates that an understanding of prey ecology can be leveraged to elucidate basic information about habitat use and resource partitioning in poorly known taxa through studies of their diets.

### Resource partitioning among *Alepisaurus*, *Anoplogaster*, and *Omosudis*

As is the case for many deep-sea fauna, reports on the foraging ecology of *An. cornuta* and *O. lowii* are limited to few diet observations and estimates of trophic position (TP) and relative feeding depths inferred from stable carbon and nitrogen isotope data. Stomach content analysis provides direct observations of trophic linkages, but the data represent a snapshot of diet and low sample sizes can result in incomplete or mischaracterized feeding habits. Stable isotope analysis (SIA) reflects more integrated feeding signals and can provide useful descriptions of general feeding guilds (e.g., micronektivores that consume fauna ~ 20–200 mm, zooplanktivores that primarily consume fauna < 20 mm), especially when samples are limited, but provide a low-resolution picture of food web structure. When many samples are available, individual diet snapshots are integrated into a finer-resolution picture of feeding habits that can be used to contextualize SIA and provide an overall broader understanding resource partitioning.

Juvenile *Al. ferox*, juvenile *An. cornuta*, and *O. lowii* have distinct overlap in forage resources that is partitioned through differential vertical habitat use and feeding strategies. All three predators consume prey with median depth habitats that correlate with two distinct depths of peak biomass in the CNP observed as epipelagic (~ 0–250 m) and mesopelagic (~ 400–750 m) acoustic scattering layers^50,51^. *Anoplogaster cornuta* consumed large quantities of small, mostly epipelagic crustaceans while *O. lowii* specialized on relatively large mesopelagic fishes, but infrequently contained more than one or two prey items in its stomach. *Alepisaurus ferox* exhibited a generalist strategy, consuming diverse intermediate-sized prey that occur throughout the water column. Although we carefully estimated foraging depths, we did not explicitly observe the depths at which each prey was captured and our method does not account for the likely consumption of prey away from their core distributions. However, our estimates generally match the known vertical habitats of all three predator species and suggest depth-stratified foraging as an important mechanism of prey partitioning.

Of the three predators, *An. cornuta* is the only species reported to perform regular diel vertical migration. Although its mesopelagic daytime habitat overlaps with the non-migratory *O. lowii*, *An. cornuta* mostly consumed prey with median depths similar to its relatively shallow nighttime distribution. Conversely, *O. lowii* only occasionally foraged on prey that predominantly occur outside of its core mesopelagic range. Some individuals fed on epipelagic prey (e.g., *Heteroteuthis hawaiiensis*, Sepiolidae) and the largest *O. lowii* in this study (270 mm SL) was directly captured on a longline (max hook depth ~ 250 m^52^). *Alepisaurus ferox* is not known to migrate on a diel cycle but is observed throughout epipelagic and mesopelagic habitats^21^. Competition with *An. cornuta* and *O. lowii* for shared prey would be reduced by foraging more evenly across the shared water column.

Predators of similar sizes had similar masses of prey in their stomachs, but almost half of the *O. lowii* stomachs were empty. Low average prey numbers and high vacuity indices are common in deep-sea fishes, consistent with infrequent feeding and low metabolic rates relative to their shallow-dwelling or migratory counterparts^5,53,54^. In deep-sea habitats, where prey densities are low, sit-and-wait foraging is the predominate feeding strategy used by fishes that consume micronekton^44^. Remotely operated vehicle observations show that both *O. lowii* (*pers. observation* EJP) and *Al. ferox*^55^ position vertically in the water column, oriented with their head up. This posture is exhibited by other mesopelagic sit-and-wait predators with forward- or lateral-facing eyes and is thought to facilitate prey detection^56,57^. *Omosudis lowii*, *Al. ferox,* and other fishes that consume micronekton also have large teeth and gapes, which are adaptations thought to increase predation success rates in food-limited habitats^44^. Fishes that consume zooplankton, including juvenile *An. cornuta*, generally have smaller teeth and actively pursue prey^54,58^. Diel migration is more energetically expensive than employing sit-and-wait strategies at depth, but there are clear benefits to feeding on higher density prey in the epipelagic^5,59,60^. Access to higher density epipelagic prey could explain the low vacuity indices of *Al. ferox* and *An. cornuta* stomachs (< 16% empty). The diverse diet of *A. ferox* reflects not just generalist feeding, but also flexibility in the behaviors employed to capture prey.

### Juvenile *Anoplogaster* specialize on hyperiid amphipods

*Anoplogaster cornuta* is typically considered to be a generalist^4,54^. However, we describe a more specialist feeding strategy in juvenile *An. cornuta,* feeding predominantly on hyperiid amphipods. Many stomachs were full of epipelagic crustaceans, similar to those “generally greatly extended by quantities of larval crustaceans” described in Mead^26^ (n = 14, 13.7–88.0 mm SL), but in a few cases instead contained only mesopelagic fish. Persistent supplementation of epipelagic forage with mesopelagic prey supports the hypothesis put forth by Romero-Romero et al.^7^ to explain increases in stable nitrogen isotope composition (δ^15^N values) with foraging depth for migratory animals relative to their epipelagic, non-migratory counterparts.

*Anoplogaster cornuta* exhibits ontogenetic descent between juvenile (< 24 mm SL) and larger subadult individuals (> 77 mm SL)^27^. Diet descriptions of individuals > 80 mm SL are limited; a total of five individuals from three combined reports suggest *An. cornuta* becomes more piscivorous as an adult^33,61,62^. The ontogeny of its dentition and gill raker morphology tracks the proposed transition away from a zooplanktivorous diet; the large, eponymous fangs begin to develop in the upper jaw at 53 mm SL and gill rakers develop into short spikes better-suited for retention of larger prey^58^. Studies using δ^15^N to estimate trophic position (TP) of sub-adult and adult *An. cornuta* consistently report a TP of ~ 3.5, reflecting a mixed diet of micronekton and zooplankton^6,62,63^. Richards et al.^33^ observed an ontogenetic increase in TP from ~ 3 to ~ 4 across individuals 84–148 mm SL in the Gulf of Mexico (GOM) that may reflect a transition from crustacean zooplankton to micronektonic fish diets.

### *Omosudis* maintains a consistent feeding strategy with size

Diets of *O. lowii* were dominated by non-migratory *Sternoptyx* spp., with sporadic consumption of lanternfishes (Myctophidae), fire squids (Pyroteuthidae), and enope squids (Enoploteuthidae) that migrate between upper mesopelagic and epipelagic habitats. Our findings are consistent with observations from the North Atlantic of *O. lowii* feeding almost exclusively on fishes and squids^32,61^, with *Sternoptyx* spp. being the most common fish prey reported by Rofen^13^. The smallest post-larvae of *O. lowii* are observed in the epipelagic, but juveniles 5–9 mm SL rapidly descend to the adult depth range > 600 m^13,31^. We observed a positive relationship between foraging depth and *O. lowii* size, but the relationship is weak, driven by shallow feeding in a few of the smallest individuals. Examination of additional small individuals is necessary to resolve whether the observed ontogenetic descent in foraging depth at small sizes is robust. Increased sample size would also improve our assessment of overall diet diversity, especially with respect to uncommon prey. However, the rate of diversity accumulation was very low over a broad region and time period (Fig. S2, Table S3), suggesting that our analysis likely captures the dominant prey and feeding strategy of *O. lowii* despite a low sample size relative to the other predator species.

In the GOM, Richards et al.^33^ observed no variability in δ^15^N values with *O. lowii* body size (36–260 mm SL), which could be influenced by prey size, identity, and habitat depth. Although we observed an increase in average prey size, we did not observe variability in the average size of the dominant prey species (*Sternoptyx* spp., 0.97 ± 0.45 g, 26.82 ± 5.42 mm SL), nor variability in the relative contributions of cephalopods and fish prey with size of *O. lowii* (43–270 mm SL). Thus, consistency in δ^15^N values across sizes reported by Richards et al.^33^ likely reflects limited ontogenetic variability in foraging ecology.

### Ontogenetic descent in *Alepisaurus*

Diets of juvenile *Al. ferox* were similar to larger individuals from the central North Pacific; 95% of all prey identified here were also reported in Portner et al.^17^ and novel prey types were mostly small fishes and crustaceans. Habitat depth generally increases with size across deep-sea taxa (e.g., Pearcy et al.^64^, Young^65^), but we observed no clear variability in the estimated foraging depth of individuals ~ 1–500 g, even as prey size increased with predator size. It is also possible that our methods of estimating foraging depth by prey size class could be refined to better capture finer scale variability in foraging depths that might reflect more continuous ontogenetic descent with size in some prey species. For individuals ~ 0.5 – 8 kg, we observed a positive trend in mean foraging depth. This ontogenetic increase in estimated foraging depth is correlated with diet variability between individuals greater and less than 1.82 kg^17^ and increases in δ^15^N values spanning two TPs for individuals across similar size ranges in the central and western Pacific Ocean^63,66^. Little is known about the life history of *Al. ferox*, but a histological study by Gibbs^28^ described 17 individuals 43–109 cm FL (0.19–2.50 kg) as “immature”. Further examination of a single “large” specimen from the same collection described mature, inactive ovaries containing Stage 3 ova^67,68^. The onset of rapid ontogenetic descent in *Al. ferox* at ~ 0.5 kg could reflect a stepwise change in depth habitat between life stages more similar to that observed in some cephalopod species^65^.

### Competitors in shared feeding grounds become prey

Variability in the consumption of each predator by *Al. ferox* can be explained by ontogenetic differences in the degree of diet and habitat overlap. *Alepisaurus ferox* has high intraspecific resource and habitat overlap, and cannibalism is most frequent across all sizes, followed by the consumption of *An. cornuta* and *O. lowii*. Given its prevalence in the diets of numerous pelagic predators^69^ and high catch rates in longline fisheries^16^, it is likely that *Al. ferox* also has higher total biomass than *An. cornuta* and *O. lowii* in the CNP. Juvenile* Al. ferox* and *An. cornuta* have intermediate overlap in foraging depths, but their comparatively low diet overlap increases with size as the size spectra of their prey converge. *Anoplogaster cornuta* > 80 mm SL were absent from *Al. ferox* stomachs. This size correlates with an ontogenetic change in coloration from the silvery gray of juveniles to the black or dark brown of adults^26,30^, as well as the putative transition to piscivory. Associated ontogenetic changes in the degree of competition or detectability may explain the absence of adult *An. cornuta*. Despite having the highest diet overlap, *Al. ferox* does not begin to consume *O. lowii* at appreciable frequencies until their mean foraging depths also overlap.

The vacuity indices of *An. Cornuta* and *O. lowii* are much lower here than previously reported for individuals sampled with nets^32,33^. Higher prey incidence in these stomachs could indicate that the predator was consumed at or near the time of feeding. The dominant crustacean (*Phrosina semilunata*) and fish (*Sternoptyx* spp.) prey taxa among all three predators are known to form relatively dense aggregations or schools^70–73^ and were sometimes found in exceptionally high numbers in *Al. ferox* stomachs (*P. semilunata,* max n = 86; *Sternoptyx* spp., max n = 124). Even if the absolute concentrations of these aggregations in the CNP are low compared to higher productivity regions, locally dense prey patches may ‘aggregate’ pelagic predators throughout the water column^74–76^. Considering the low vacuity indices, increased consumption of *An. cornuta* and *O. lowii* with increased overlap in diet and foraging depth suggests the act of feeding on prey aggregations might increase the rates at which large *Al. ferox* encounters these three deep-sea fishes.

### Future directions

This work highlights our ability to harness pelagic predators as biological samplers to address critical gaps in our understanding of deep-sea food web structure. Expanding this type of work to other pelagic predators that are captured by longlines (e.g., snake mackerels and barracudinas) would increase the diversity of prey taxa that could be sampled. Collaboration with fisheries observer programs allows for much higher spatial and temporal resolution sampling than is possible with ship-based scientific exploration. Long term monitoring of pelagic predator diets would greatly facilitate fundamental biogeographic descriptions of common deep-sea taxa and their foraging ecologies, as well as large-scale studies of the responses of pelagic prey communities to environmental perturbations.

### Supplementary Information

Supplementary Figures and Tables.

Supplementary Table S1.

Supplementary Table S2.

Supplementary Data File S1.

## Data Availability

The data required to replicate the analyses presented in this study are available as supplemental material. See “Read me” sheet in Supplementary Data File S1 for a description of the data provided for each figure/analysis.
