# Supplementary material for: Harnessing a mesopelagic predator as a biological sampler reveals taxonomic and vertical resource partitioning among three poorly known deep-sea fishes
Source: Sci Rep. 2023 Sep 26;13:16078. doi: 10.1038/s41598-023-41298-9 (PMC10522621; doi:10.1038/s41598-023-41298-9)
Supplement: Supplementary file 1 — Supplementary Figures and Tables. [file 41598_2023_41298_MOESM1_ESM.pdf]

Harnessing a mesopelagic predator as a biological sampler reveals taxonomic and vertical resource partitioning among three poorly known deep-sea fishes

Elan J. Portner[1*], Tor Mowatt-Larssen[2], Alejandro Cano-Lasso Carretero[1], Emily A. Contreras[3], Phoebe A. Woodworth-Jefcoats[3], Benjamin W. Frable[1], C. Anela Choy[1]

[1] Scripps Institution of Oceanography, University of California San Diego, La Jolla, CA USA

[2] Virginia Institute of Marine Science, William & Mary, Gloucester Point, VA USA

[3] Cooperative Institute for Marine and Atmospheric Research, University of Hawai‘i, Honolulu, HI USA

[4] Pacific Islands Fisheries Science Center, National Marine Fisheries Service, National Oceanic and Atmospheric Administration, Honolulu, HI USA

*corresponding author: (email: eportner@ucsd.edu)

Supporting information

This document contains Supplementary Figures S1, S2, S3, S4, and S5; captions for Supplementary Tables S1 and S2 (tables given in a separate excel files); and Supplementary Tables S3, S4, S5, and S6.

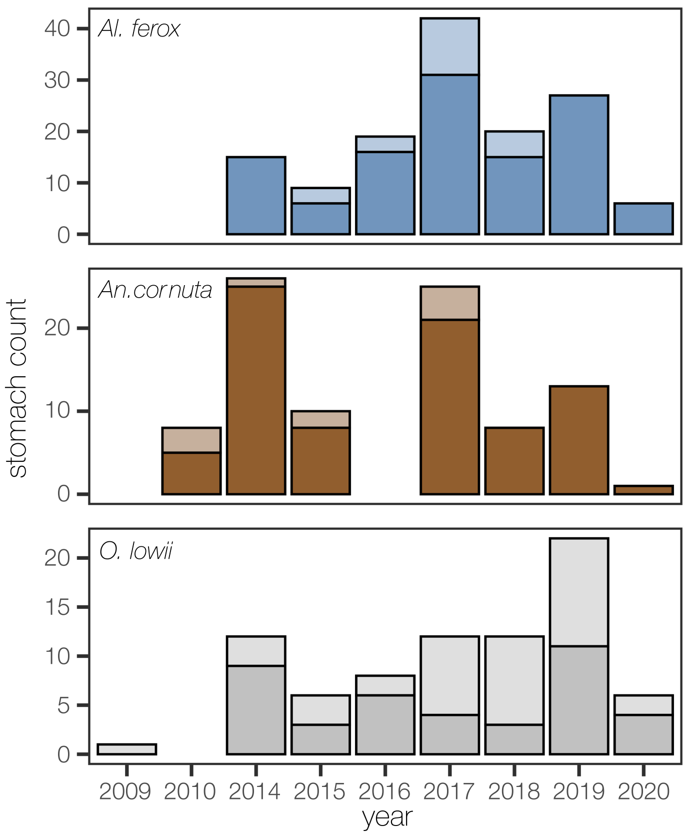

Fig. S1: Number of specimens dissected by collection year for each predator species. In all panels, darker shading indicates stomachs with prey and lighter shading indicates empty stomachs.

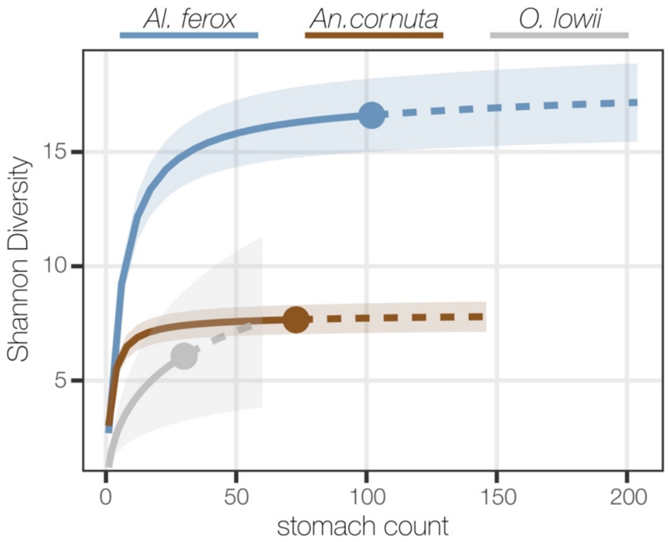

Fig. S2: Diversity accumulation curves describing rate of family-level prey discovery quantified as Shannon Diversity (hill number of order q =1). Solid lines indicate interpolated diversity up to the sample size (circles) and dashed lines indicate extrapolated diversity to two-times the sample size.

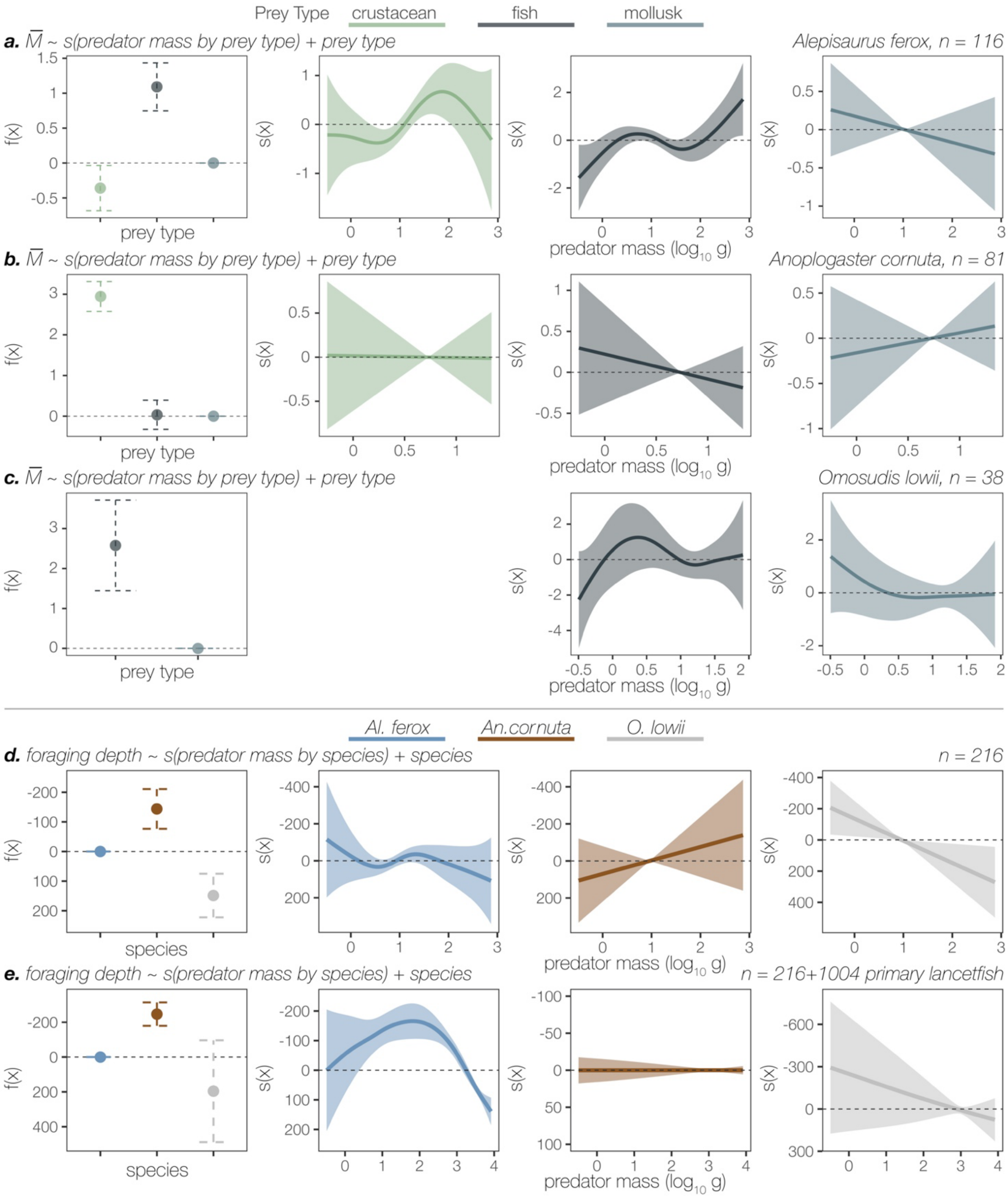

Fig. S3: Partial-effects plots from generalized additive models describing changes in the proportional mass ($\bar{M}$) of broad prey types with predator size (***a, b, c***) and the effects of predator species and mass on estimated foraging depth (***d, e***). Plots describe the relationship between each covariate and its parametric contribution ("f(x)") or the contribution of its smoother ("s(x)") to the model's fitted values. See Table 2 for full model summaries.

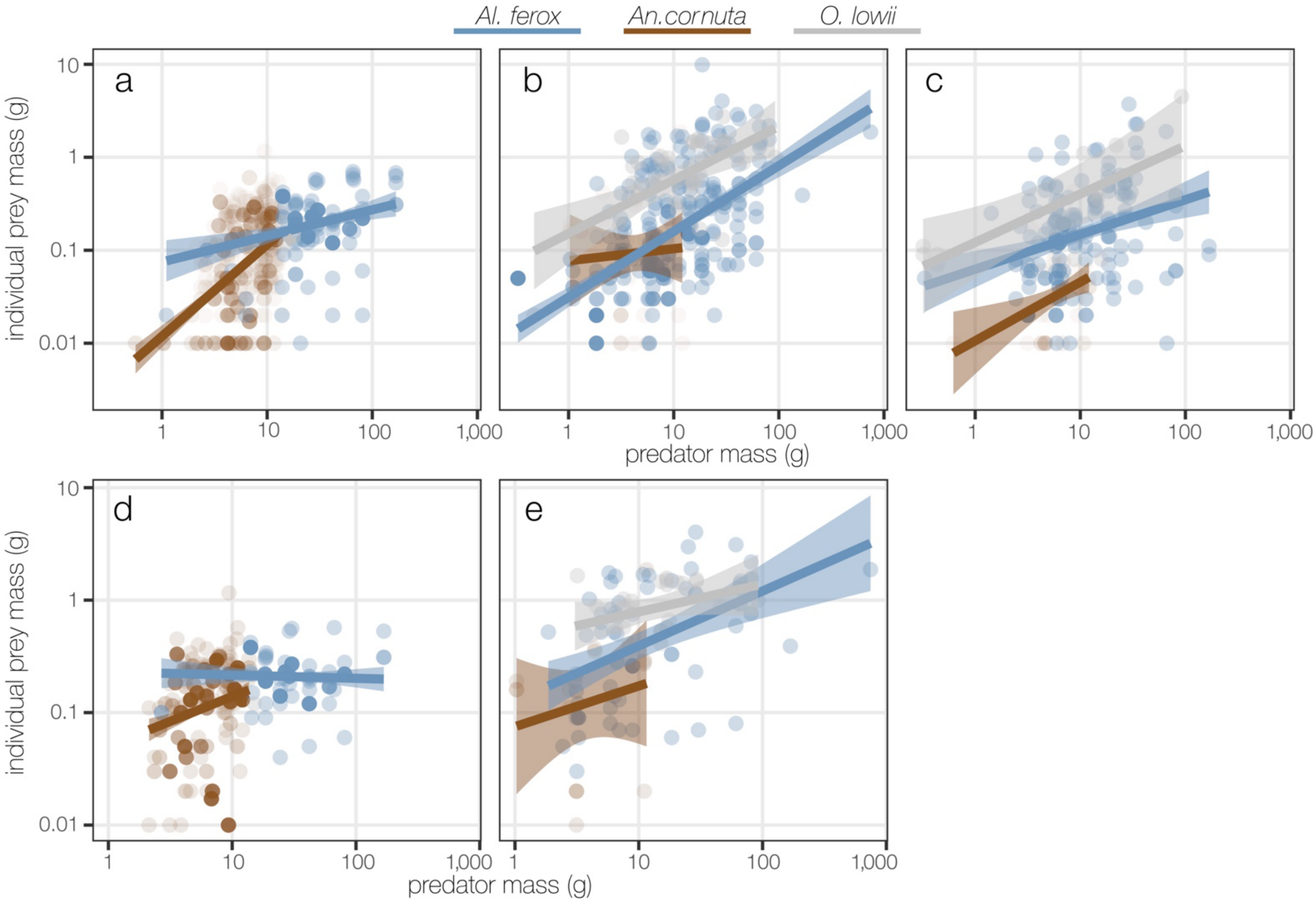

Fig. S4: Linear models describing the effects of predator species and mass on individual prey mass for broad prey types (crustaceans (**a**), fishes (**b**), molluscs (**c**)) and the most abundant prey families (Phrosinidae (**d**), Sternoptychidae (**e**)). Axes are on the log_{10}-scale. ANCOVA results for each model are given in Table S6.

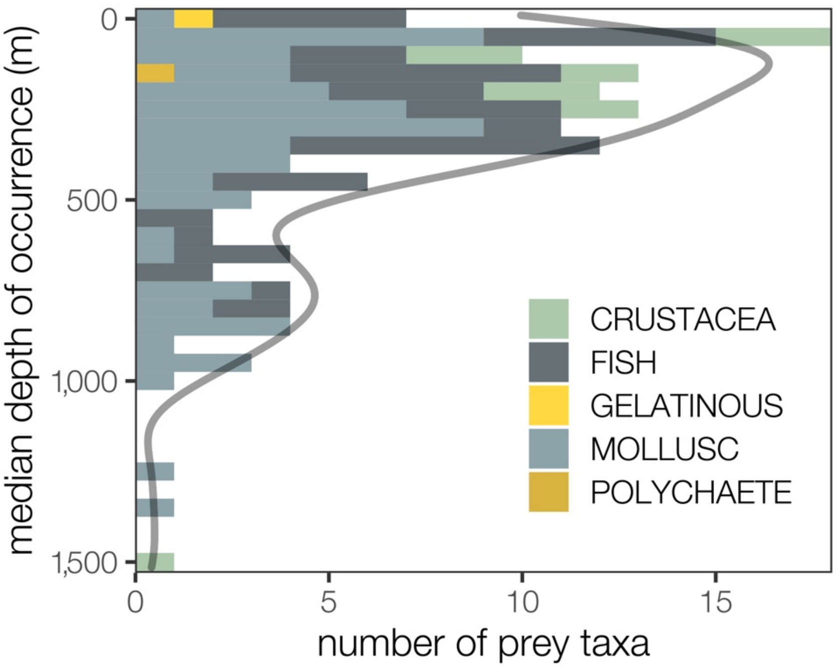

Fig. S5: Median depth assignments for all unique prey taxa colored by prey type. Some taxa are represented by multiple size classes with different depth habitats, see Table S1. Overlaying density plot summarizes the bimodal distribution of median depths of occurrence.

Table S1: Median depth assignments for each prey taxa (per size class when appropriate). See READ_ME and data tables in "Supplementary_TableS1.xlsx".

Table S2: Contributions of each unique prey taxa to the diets of juvenile *Alepisaurus ferox*, juvenile *Anoplogaster cornuta*, and *Omosudis lowii* are given for each predator as the total number (*n*), mean proportional abundance ($\bar{N}$), total mass (*m*), mean proportional mass ($\bar{M}$), frequency (*FO*) and percent frequency of occurrence (*% FO*). These metrics are also summarized at the prey type level for each species. See "Supplementary_TableS2.xlsx".

Table S3: Observed Shannon Diversity (^{1}D) and estimated true diversity ($^{1}D_{ex}$ (± se)) quantified using family-level diversity accumulation curves (see Fig. S2). The sample size included in the analysis (*n*) and the average sample size required to observe 95% of the estimated true diversity (95% coverage, $t_{95\%}$) are also given.

	n	^{1}D	$^{1}D_{ex}$ **(± se)**	$t_{95\%}$
Alepisaurus ferox	102	16.61	17.57 (0.82)	34
Anoplogaster cornuta	73	7.67	7.88 (0.37)	12
Omosudis lowii	30	6.07	9.33 (2.62)	113

Table S4: Mean pairwise Morisita-Horn Similarity for all within- and between group comparisons are given in the lower half of table (gray shading). Test statistics from PERMANOVA and PERMDISP are given in the upper half (*t*-value PERMANOA, *t*-value PERMDISP). The p-values for all comparisons are 0.001 unless otherwise indicated ("*" = $0.001 < p < 0.01$, "**" = $0.01 < p < 0.05$).

	Alepisaurus ferox	***Anoplogaster cornuta***	***Omosudis lowii***
Alepisaurus ferox	17.60	(6.25, 10.10)	(2.23, 4.31*)
Anoplogaster cornuta	13.28	56.48	(6.99, 2.53**)
Omosudis lowii	22.44	6.71	39.85

Table S5: Summaries for multilinear models describing the effects of predator species and mass on individual prey mass and the total count and mass of prey per stomach (see Fig. 3). Model results are given as the Adjusted R^2 (*adj. R^2*), *F*-statistic, an *p*-value. The estimate, standard error (*se*), *t*-values (*t*), and p-values (*p*) are given for all terms and their interactions (*:*) for each model.

a. *log_{10}(individual prey mass) ~ log_{10}(predator mass)*species*	*Adj. Multiple R^2* = 0.30; *F*(5, 1602) = 137.86; *p* < 2.2 E^{-16}			
	Estimate	***se***	***t***	***p***
(Intercept)	-1.84	0.06	-32.92	2.00 E^{-16}
log_{10}(predator mass)	0.86	0.07	12.68	2.00 E^{-16}
Alepisaurus vs. Anoplogaster	0.46	0.07	6.57	6.72 E^{-11}
Omosudis vs. Anoplogaster	0.98	0.14	6.98	4.43 E^{-12}
Alepisaurus vs. Omosudis	-0.52	0.14	-3.82	1.37 E^{-04}
log_{10}(predator mass):Alepisaurus vs. log_{10}(predator mass):Anoplogaster	-0.34	0.08	-4.36	1.38 E^{-05}
log_{10}(predator mass):Omosudis vs. log_{10}(predator mass): Anoplogaster	-0.31	0.13	-2.31	0.02
log_{10}(predator mass):Alepisaurus vs. log_{10}(predator mass): Omosudis	-0.03	0.12	-0.21	0.83
b. *log_2(prey count) ~ log_{10}(predator mass)*species*	*Adj. Multiple R^2* = 0.32; *F*(5, 231) = 23.57; *p* < 2.2 E^{-16}			
	Estimate	***se***	***t***	***p***
(Intercept)	1.94	0.38	5.11	2.37 E^{-07}
log_{10}(predator mass)	1.54	0.48	3.23	2.62E^{-03}
Alepisaurus vs. Anoplogaster	-0.24	0.46	-0.52	0.50
Omosudis vs. Anoplogaster	-1.64	0.57	-2.91	2.16 E^{-03}
Alepisaurus vs. Omosudis	1.41	0.49	2.88	4.34 E^{-03}
log_{10}(predator mass):Alepisaurus vs. log_{10}(predator mass):Anoplogaster	-1.01	0.52	-1.92	0.076
log_{10}(predator mass):Omosudis vs. log_{10}(predator mass): Anoplogaster	-1.36	0.60	-2.27	3.61 E^{-02}
log_{10}(predator mass):Alepisaurus vs. log_{10}(predator mass): Omosudis	0.36	0.43	0.85	0.40
c. *log_{10}(total prey mass) ~ log_{10}(predator mass)*species*	*Adj. Multiple R^2* = 0.46; *F*(5, 231) = 41.78; *p* < 2.2 E^{-16}			
	Estimate	***se***	***t***	***p***
(Intercept)	-1.00	0.14	-7.26	5.96 E^{-12}
log_{10}(predator mass)	1.30	0.17	7.47	1.62 E^{-12}
Alepisaurus vs. Anoplogaster	0.22	0.17	1.31	0.19
Omosudis vs. Anoplogaster	0.19	0.20	0.95	0.34
Alepisaurus vs. Omosudis	0.02	0.18	0.13	0.89
log_{10}(predator mass):Alepisaurus vs. log_{10}(predator mass):Anoplogaster	-0.44	0.19	-2.34	0.02
log_{10}(predator mass):Omosudis vs. log_{10}(predator mass): Anoplogaster	-0.66	0.22	-3.03	2.72 E^{-03}
log_{10}(predator mass):Alepisaurus vs. log_{10}(predator mass): Omosudis	0.22	0.15	1.41	0.16

Table S6: Model results and ANCOVA outputs for all linear models in Fig. S3 describing effect of predator species and mass on prey mass for each main prey group (***a-c***) and the two most abundant prey families (***d,e***). The sum of squares (*SS*), degrees of freedom (*df*), F-statistics (*F*) and p-values (*p*) are given for terms and their interactions (*:*) for each model.

log10(individual prey mass)~log10(predator mass)*species				
	SS	***df***	***F***	***p***
a. *CRUSTACEANS*		$F(5, 952) = 64.30$, $p < .001$, *adj.* $R^2 = 0.25$		
Intercept	210.67	1	1124.85	< 2.20 E^{-16}
log$_{10}$(predator mass)	37.64	1	200.98	< 2.20 E^{-16}
species	4.12	2	11.01	1.88 E^{-05}
log$_{10}$(predator mass):species	5.51	2	14.71	5.09 E^{-07}
Residuals	178.30	952		
b. *FISHES*		$F(5, 396) = 50.53$, $p < .001$, *adj.* $R^2 = 0.38$		
Intercept	183.63	1	751.00	< 2.20 E^{-16}
log$_{10}$(predator mass)	43.63	1	178.44	< 2.20 E^{-16}
species	3.08	2	6.29	2.05 E^{-03}
log$_{10}$(predator mass):species	1.09	2	2.23	0.11
Residuals	96.83	396		
c. *MOLLUSCS.*		$F(5, 237) = 25.79$, $p < .001$, *adj.* $R^2 = 0.34$		
Intercept	19.46	1	90.62	< 2.20 E^{-16}
log$_{10}$(predator mass)	1.36	1	6.33	0.01
species	3.45	2	8.03	4.23 E^{-04}
log$_{10}$(predator mass):species	0.29	2	0.67	0.51
Residuals	50.88	237		
d. *Phrosinidae*		$F(3, 563) = 24.65$, $p < .001$, *adj.* $R^2 = 0.11$		
Intercept	38.50	1	304.51	< 2.20 E^{-16}
log$_{10}$(predator mass)	3.21	1	25.35	6.45 E^{-07}
species	1.66	1	13.09	3.24 E^{-04}
log$_{10}$(predator mass):species	1.35	1	10.67	1.16 E^{-03}
Residuals	71.18	563		
e. *Sternoptychidae*		$F(5, 101) = 11.10$, $p < .001$, *adj.* $R^2 = 0.32$		
Intercept	5.97	1	27.69	0.29
log$_{10}$(predator mass)	0.28	1	1.28	0.26
species	0.84	2	1.94	0.15
log$_{10}$(predator mass):species	0.15	2	0.35	0.70
Residuals	21.79	101		
